# Viruses in reptiles

**DOI:** 10.1186/1297-9716-42-100

**Published:** 2011-09-21

**Authors:** Ellen Ariel

**Affiliations:** 1 Microbiology and Immunology, School of Veterinary and Biomedical Sciences, James Cook University, Townsville, Queensland 4810, Australia

## Abstract

The etiology of reptilian viral diseases can be attributed to a wide range of viruses occurring across different genera and families. Thirty to forty years ago, studies of viruses in reptiles focused mainly on the zoonotic potential of arboviruses in reptiles and much effort went into surveys and challenge trials of a range of reptiles with eastern and western equine encephalitis as well as Japanese encephalitis viruses. In the past decade, outbreaks of infection with West Nile virus in human populations and in farmed alligators in the USA has seen the research emphasis placed on the issue of reptiles, particularly crocodiles and alligators, being susceptible to, and reservoirs for, this serious zoonotic disease. Although there are many recognised reptilian viruses, the evidence for those being primary pathogens is relatively limited. Transmission studies establishing pathogenicity and cofactors are likewise scarce, possibly due to the relatively low commercial importance of reptiles, difficulties with the availability of animals and permits for statistically sound experiments, difficulties with housing of reptiles in an experimental setting or the inability to propagate some viruses in cell culture to sufficient titres for transmission studies. Viruses as causes of direct loss of threatened species, such as the chelonid fibropapilloma associated herpesvirus and ranaviruses in farmed and wild tortoises and turtles, have re-focused attention back to the characterisation of the viruses as well as diagnosis and pathogenesis in the host itself.

1. Introduction

2. Methods for working with reptilian viruses

3. Reptilian viruses described by virus families

3.1. Herpesviridae

3.2. Iridoviridae

3.2.1 Ranavirus

3.2.2 Erythrocytic virus

3.2.3 Iridovirus

3.3. Poxviridae

3.4. Adenoviridae

3.5. Papillomaviridae

3.6. Parvoviridae

3.7. Reoviridae

3.8. Retroviridae and inclusion body disease of Boid snakes

3.9. Arboviruses

3.9.1. Flaviviridae

3.9.2. Togaviridae

3.10. Caliciviridae

3.11. Picornaviridae

3.12. Paramyxoviridae

4. Summary

5. Acknowledgements

6. Competing interests

7. References

## 1. Introduction

The etiology of reptilian viral diseases can be attributed to a wide range of viruses occurring across different genera and families. Thirty to forty years ago, studies of viruses in reptiles focused mainly on the zoonotic potential of arboviruses in reptiles and much effort went into surveys and challenge trials of a range of reptiles with eastern and western equine encephalitis as well as Japanese encephalitis viruses [1-3]. In the past decade, outbreaks of infection with West Nile virus in human populations and in farmed alligators in the USA have seen the research emphasis placed on the issue of reptiles, particularly crocodiles and alligators, being susceptible to, and reservoirs for, this serious zoonotic disease [4-7]. Although there are many recognised reptilian viruses, the evidence for those being primary pathogens is relatively limited. Transmission studies establishing pathogenicity and cofactors are likewise scarce, possibly due to the relatively low commercial importance of reptiles, difficulties with the availability of animals and permits for statistically sound experiments, difficulties with housing of reptiles in an experimental setting or the inability to propagate some viruses in cell culture to sufficient titres for transmission studies. Viruses as causes of direct loss of threatened species, such as the chelonid fibropapilloma associated herpesvirus and ranaviruses in farmed and wild tortoises and turtles, have re-focused attention back on the characterisation of the viruses [8,9] as well as diagnosis and pathogenesis in the host itself [10-13].

## 2. Methods for working with reptilian viruses

Generally, diagnosis of reptilian viruses can be approached like all other viruses. Histopathology can give the initial indication of a viral infection and most infections are described alongside the pathological changes they induce. Some viruses have been isolated, characterized and used in challenge trials or to produce specific antisera for immunological tests, but like all other fields of virology, reptilian virus researchers are venturing into both serological surveys and molecular tools for diagnosing viral infections [8,14-20].

Isolation of viruses in culture has the advantage of amplifying the agent for easier identification and characterisation, but also for use in transmission studies. Specific reptilian cell lines are available that will support viral growth and for some viruses display cytopathic effect, where others do not cause CPE or do not grow at all in known cell culture systems. For those cell lines that support propagation of a particular virus, the temperature regime for both cell lines and viral growth will be different from mammalian systems due to the poikilothermic nature of reptiles. Zoonotic viruses have been isolated from reptiles since 1939 when Rosenbusch isolated Western Equine Encephalitis from a *Bothrops alternate*, followed by Japanese encephalitis virus from snakes, calicivirus from snakes, a flavivirus-like agent from tortoises and West Nile virus from alligators using either mammalian derived cell lines at 37°C or mosquito cell lines at 28°C [7,21-25]. Several other reptilian viruses will grow in cell lines derived from mammals such as Vero cells or in cell lines derived from fish [26,27]. Clark and Karzon isolated a herpesvirus from iguana in tissue explants from an apparently healthy iguana in 1972 [28] and since then several reptilian cell culture systems have been established and are now available from the American Type Culture Collection. Paramyxovirus grows readily in cobra eggs, viper heart, gecko embryo and rattlesnake fibroma cell lines [29,30] and ranavirus will grow in a variety of cell lines including fish cell lines at a range of temperatures below 30°C [27].

Where electron microscopy (EM) used to be the ultimate diagnostic tool for viruses, polymerase chain reaction (PCR) has revolutionized our ability to identify infectious agents with generic and specific primers used for diagnosis. Subsequent sequencing of the viral genome opens possibilities for fast characterization, insight into their phylogenetic relationship and epidemiological investigations. The drastic decrease in the cost of sequencing has turned the diagnostic attention to molecular methods, which has the added advantage of accurate diagnosis and the subsequent possibility of reproducing the antigenic part of the virus, irrespective of our ability to culture the viral pathogen in vitro. However electron microscopy still has a diagnostic role since agents identified under the EM scope may direct the diagnostician toward selecting the best primers to use.

With the current trend and affordable sequencing costs, researchers will keep looking for and finding viruses in reptiles at a great rate and over the next decade we are likely to see the clades being populated with new findings, known isolates being placed into taxonomic relationships and possibly revealing genetic virulence markers.

## 3. Reptilian viruses described by virus families

The type of genome and the presence of an envelope are important in the replication strategy of viruses and subsequently the possibilities of diagnosis, prevention and control of the associated disease. The viral families in this review are presented with like groups as indicated in Table 1, and not in order of importance.

**Table 1:** List of virus families of importance to reptile health with genome and presence of envelope indicated

Virus family	Genome	Envelope
Herpesviridae	Ds DNA	+

Iridoviridae	Ds DNA	+

Poxviridae	Ds DNA	+

Adenoviridae	Ds DNA	-

Papillomaviridae	Ds DNA	-

Parvoviridae	Ss DNA	-

Reoviridae	Ds RNA	-

Retroviridae	Pos ss RNA	+

Flaviviridae	Pos ss RNA	+

Togaviridae	Pos ss RNA	+

Caliciviridae	Pos ss RNA	-

Picornaviridae	Pos ss RNA	-

Paramyxoviridae	Neg ss RNA	+

Rhabdoviridae	Neg ss RNA	+

### 3.1. Herpesviridae

Herpesvirus infections appear to manifest as acute signs which may turn latent and be quiescent for the rest of the animal's life, or until the host becomes sufficiently stressed for the virus to reappear as a disease [31]. Reptilian herpesviruses fall into the family *Herpesviridae *together with mammalian and avian herpesviruses. Chelonid herpesvirus 5 and 6 in marine turtles are unassigned species in the Subfamily *Alphaherpesvirinae *and although other reptilian herpesviruses are still unassigned by the International Committee on Taxonomy of Viruses (ICTV), molecular characterisation of available isolates places them within the Subfamily *Alphaherpesvirinae *in the proposed genus *Chelonivirus *[32-34]. Originally herpesviruses were classified according to their host, disease signs and morphology as determined by EM, where the current methods are based on sequencing of the viruses followed by phylogenetic analysis. Because many of the viruses described prior to development of these methods have not or cannot be sequenced, there is some confusion as to their taxonomic position. This review will use the classification of chelonian herpesviruses based on sequence analysis as proposed by Bicknese et al. [34] with the ICTV classification in brackets when known.

Chelonid fibropapilloma-associated herpesvirus (CFPHV) (Chelonid herpesvirus 5), is associated with the development of fibropapillomas and fibromas in marine turtles in all tropical waters, both externally on the epidermis, eyes, carapace and plastron, and in severe cases on the serosal surface of internal organs [35-40]. Fibropapillomatosis (FP) is a major chronic disease of juvenile green turtles and was considered the most significant cause of strandings and mortality in waters around Florida [41] and Hawaii [42]. An infection by fibropapillomatosis herpesvirus appears to be associated with oncogenesis under certain circumstances and considerable research effort has focused on the resultant disease: fibropapillomatosis of marine turtles [8,13,39,41,43-45].

Although many biotic factors such as leeches, mites, other viruses and algal blooms as well as abiotic environmental factors and adjacent land use are associated with FP of wild turtles [32,34,46-49], the CFPHV is implicated as the etiological agent of the disease [20,43,50-52]. It is suspected to operate under certain environmental conditions and in synergy with immune system modulators which may influence the persistence and severity of the lesions [38,40,44,53,54]. Wildlife and environmental management agencies are particularly concerned due to the potential population impact this disease could exert on otherwise threatened species [55].

The lung-eye-trachea disease-associated virus (LETV) (Chelonid herpesvirus 6), was first described in 1 year old green turtles raised in mariculture. As the name implies, the virus affects the eyes and respiratory tract of the turtles and has a clinical course of 2-3 weeks. The virus was cultured in green turtle kidney cells and initially visualized by EM [56]. The ability of LETV to grow in vitro has enabled further study of this virus and the development of serological tools to detect previous exposure to the virus in turtles [57,58].

Grey Patch Disease (GPD)-associated virus (Chelonid herpesvirus 1) affecting cultured green turtle hatchlings (*Chelonia mydas*) was reported to cause mortalities in 5-20% of the severely afflicted animals [59]. The disease appeared as circular papular skin lesions coalescing into diffuse grey skin lesions with superficial epidermal necrosis and can affect 90-100% of all hatchlings. The lesions were characterised by hyperkeratosis and hyperplasia with acanthosis. Epidermal cells displayed basophilic intranuclear inclusions and marginated chromatin. Intranuclear enveloped particles of 160-180 nm with an electron dense core of 105-120 nm were visualised by EM. Transmission is thought to be vertical or water-borne [59]. Sudden changes in water temperature could bring about the onset of symptoms in tank reared turtles [60], with low water temperatures leading to a longer lasting but less severe disease than high water temperatures [61].

Two herpesviruses from loggerhead turtles (*Caretta caretta*) were identified by PCR in lesions of moribund animals [32]. One virus was termed the "loggerhead genital-respiratory herpesvirus" (LGRV), and the other the "loggerhead orocutaneous herpesvirus" (LOCV) according to the lesions they were identified from. Both viruses were thought to be opportunistic in debilitated turtles.

Tortoise Herpesviruses

Herpesviruses are now commonly identified in tortoises of zoological collections due to improved diagnostic methods and several strains have been described and classified into tortoise herpesviruses 1 to 4 (THV1-4) [34,62-64]. Pathological changes caused by herpesvirus infections in tortoises include hepatitis, stomatitis, respiratory tract infection, conjunctivitis and central nervous system involvement [65-70].

Elapid herpesvirus (Indian cobra herpesvirus) was associated with degeneration and focal necrosis of columnar glandular epithelial cells in the venom gland of Siamese cobras (*Naja naja kaouthia*) with reduced venom production [71].

Herpesviruses have also been detected in lizards: Green lizard herpesvirus was identified in Green lizard papillomas. It was accompanied by two other viruses [72], and may have been an incidental finding of an otherwise latent infection. Herpesviruses were also identified in lizards with stomatitis [73,74] and were named *Varanid herpesvirus 1 *- or gerrhosaurid herpesviruses 1-3. Iguanid herpesvirus 1 was isolated during routine tissue explants from the spleen, kidney and heart of a normal adult *Iguana iguana *[28].

### 3.2. Iridoviridae

#### 3.2.1 Ranavirus

*Ranavirus *is a genus in the *Iridoviridae *family represented by the type species Frog Virus 3 (FV3). Previous accounts of iridovirus causing systemic infections were later classified as ranavirus in many cases. Ranavirus has received increasing attention due to its involvement in serious diseases of fish and amphibians to the extent that epizootic haematopoietic necrosis virus (EHNV) in fish and ranavirus in general for amphibians meet the criteria for listing by the World Organization for Animal Health (OIE) [75]. Ranaviruses have been implicated in the world-wide decline of amphibians and mass mortalities in both aquaculture and wild fish stock. Some of these isolates are very similar on a serological and molecular level [76-80] and have been reported to be able to infect hosts across classes [10,81,82]. This ability of the virus has the potential to compromise prevention and control measures, since amphibians, reptiles and fish may act as reservoir species for each other.

Until recently, overt diseases in reptiles caused by systemic iridovirus were only rarely reported: namely in a spur-tailed Mediterranean land tortoise, *Testudo hermanni *[83] and a gopher tortoise (*Bopherus polyphemus*) [84]. However, with increased awareness, several instances of severe mortality events in reptiles associated with ranavirus infections have been described in the last decade in both zoological collections [9,85-87], free-ranging [10], and farmed reptiles [88]. Retrospective studies of archival material may bring even more cases to light. Challenge studies were carried out on red eared sliders (*Trachemys scripta elegans*) with a ranavirus isolate from Burmese Star tortoise. The red eared sliders developed clinical signs and lesions consistent with those observed in the Burmese tortoises. Virions were observed in lesions by EM and ranavirus was re-isolated from challenged animals [89].

Ranavirus infections of reptiles appear to target multiple organs including the stomach, oesophagus, lungs, spleen, liver and kidney, although some isolates may have a propensity for infecting the respiratory tract [10,85,87]. Intracytoplasmic inclusion bodies can be identified in some cases and in addition to virus isolation in established cell lines [27], diagnosis is made on the basis of immuno assays and PCR targeting the major capsid protein or the polymerase gene [78,87,90]. Benetka et al., [91] reported co-infection with ranavirus and chelonid herpesvirus in a severely ill leopard tortoise. The diagnosis was made by PCR from pharyngeal and oral swabs, but the tortoise was also compromised by a mixed bacterial infection and recovered after antibiotic treatment, showing that ranavirus may have been a co-infection or a pre-disposing factor rather than the primary cause of the disease. Ranaviruses are easily cultured in vitro and their genome has been studied extensively [78,82,90,92].

#### 3.2.2 Erythrocytic virus

Cytoplasmic inclusions in erythrocytes of amphibians, fish and reptiles have long been known to be of iridoviral origin due to light and EM [93-96]. The infection could possibly contribute to anaemia in the host although the clinical significance is not clear. Recently, Wellehan et al. characterised an erythrocytic iridovirus by means of molecular techniques and found that it falls in a group all of its own within the Iridoviridae and may represent a new genus [97].

#### 3.2.3 Iridovirus

Although viruses in this genus mostly occur in invertebrates, they do occasionally occur in reptiles [98] and may contribute to disease, but the latter is not clear. Viruses from this genus are, however, often isolated from the prey animals of reptiles, for example commercially grown crickets fed to lizards, and could be incidental residents in the reptile hosts rather than actually infecting and replicating in them [99]. They do grow in reptilian cell lines such as Viper Heart (VH-2) and Terrapene Heart (TH-1) which indicate that they can infect reptiles and isolates from a chameleon (*Chamaeloe hoehnelii*) were found to be highly pathogenic to crickets (*Gryllus bimaculatus*) [99] showing that reptiles may be reservoirs for an invertebrate virus.

### 3.3. Poxviridae

Poxviruses have been identified in skin lesions of *Caiman sclerops *and *Caiman crocodilus fuscus *[100-102]*Crocodylus niloticus, C. porosus *and *C. johnstoni *[103-107]. The lesions presented as brown raised ulcers on the ventral skin, the head region or in the oral cavity. Eosinophilic intracytoplasmic inclusions were observed within hypertrophied epithelial cells. At higher magnification (EM) the inclusions were seen to be viral arrays consisting of pox-like virions 100-200 nm in diameter, which is small compared to poxviruses of other vertebrates and insects. In all cases, morbidity was high, and mortality low, but due to the disfiguring nature of the disease, it is still an economically important disease for crocodile farming. A single case of poxvirus in the deep epidermal cells of a Hermann tortoise (*Testudo hermanni*) that succumbed to broncho-pneumonia [108], a co-infection with Chlamydia in a flap-necked chameleon (*Chamaeleo dilepsis*) [109] and a tegu (*Tupinambis teguixin*) with poxviral dermatitis that healed spontaneously over four months have also been reported [110], but generally, poxvirus infections of reptiles do not seem to be widespread. Molecular analysis by Afonso et al. [111] places crocodile pox virus from Nile crocodiles in a new proposed genus within the Chordopoxvirinae.

### 3.4. Adenoviridae

Adenoviruses cause respiratory infections in many vertebrate species. Infections have been diagnosed in crocodiles, [112,113], snakes [17,114-116], lizards [17,117-119] and turtles [120,121]. Infections in reptiles can be accompanied by lethargy, neurological disorder, esophagitis, hepatitis, splenitis or gastroenteritis [114,117,118,121]. Adenovirus was isolated in vitro from a lizard [17] and a Corn snake, where the subsequent cytopathic effect (CPE) observed in cell cultures included intranuclear inclusion bodies and finally cell lysis [122]. Diagnosis of adenovirus is now largely done by molecular tools such as PCR directly on swabs or organs followed by sequencing [123] or in situ hybridisation of formalin fixed tissues [124].

A great amount of work has gone into the phylogenetic analysis of adenoviruses, which has resulted in the proposal for creation of three new genera recently in addition to the *Mastadenovirus *and *Aviadenovirus *genera which traditionally covered mammalian and avian adenoviruses respectively [125]. The latest addition is *Ictadenovirus *which hosts a single member from a fish, namely the White sturgeon adenovirus 1. *Atadenovirus *was named according to the very high A+T genome base composition in some of the early viruses in this group, which encompass viruses from reptilian, avian and mammalian hosts. Until recently, all reptile adenoviruses belonged to the *Atadenovirus *genus indicating strong co-evolution of the host and virus. *Siadenoviruses *have a very small genome (30 kb) and a putative sialidase gene at the terminus of the genome, which gave rise to the name of this genus [126]. In 2009, Rivera et al. characterised an adenovirus from Sulawesi tortoises (*Indotestudo forsteni*) by sequence analysis as a novel adenovirus of the genus *Siadenovirus *which currently makes it the only reptile virus in this genus [127]. The chelonian adenovirus recently described by Farkas [121] does not fit into any of the recognized genera of the adenovirus family.

### 3.5. Papillomaviridae

Two side-necked turtles (*Platemys platycephala*) exhibited symptoms of circular papular skin lesions on the head and forelimbs [128]. Histological examination of the epidermis revealed hyperkeratosis and hyperplasia with acanthosis, but no inclusions were observed. Intranuclear crystalline arrays of hexagonal particles of 42 nm diameter were visualised by electron microscopy. The particles resembled papilloma virions similar to those seen in mammalian wart lesions [128]. Drury et al. identified papilloma-like particles in lung-washings of a Horsfield Russian tortoise [62].

Papilloma-associated viruses were also identified via EM of benign papillomas from Green lizards (*Lacerta viridis*) by Raynaud and Adrian [72]. The virions were found only in the highly keratinised regions of the papillomas and displayed morphologies similar to papillomavirus, herpesvirus and reovirus. These mixed viral infections were consistent in the three animals examined. None of the viruses were cultivated in vitro [72]. Although the etiological agent of the papillomas could not be determined with confidence, the papillomavirus was implicated because this group is often associated with papillomas in mammals [129]. The other two viruses may have been incidental findings.

Lately, papillomavirus was reported in samples from sea turtles, green turtle (*Chelonia mydas*) and loggerhead turtle (*Caretta caretta*) with fibropapillomatosis [32,130]. Herbst et al. [49] characterised the two papillomaviruses and found that they fall into a distinct chelonian clade.

### 3.6. Parvoviridae

The only parvovirus recorded in reptiles is the Dependovirus which requires the presence of an adenovirus infection for replication. This virus was found associated with an adenovirus in intestinal epithelium of several snake species [114,131-133] and in the intestinal tract and liver of a bearded dragon, *Pogona vitticeps *[119,134]. The virus was isolated from a royal python and identified as *serpentine adeno-associated virus *by Farkas et al. [133].

### 3.7. Reoviridae

Reoviruses can cause severe and often fatal disease in reptiles typically presenting as pneumonia and neurological disorder [135-137]. Reovirus were isolated from the kidney, liver and spleen of a moribund python (*Python regius*) and from the brain of a rattlesnake exhibiting neurological symptoms [26,136]. The isolates grew in iguana (IgH2) and Vero cells respectively and displayed a CPE of syncytical giant cell formation [136] typical of reptilian reovirus [138]. Lamirande et al. isolated reovirus from two dead elaphid snakes and were able to induce pneumonia and tracheitis in a black ratsnake (*Elaphe obsolete*), re-isolate the virus and induce similar symptoms in another black ratsnake [137]. A reovirus was also one of three viruses associated with papillomas in the Green lizard *Lacerta viridis *[72]. Reoviruses are fusogenic and grow readily in cell culture [138]. Identification is by PCR and subsequent genomic analysis of reptilian isolates has so far placed them all in the Orthoreovirus genus [135].

### 3.8. Retroviridae and inclusion body disease of Boid snakes

Retroviruses often appear as incidental findings with no disease reported in turtles, crocodiles, tuataras, a Komodo dragon and snakes [19,139-142]. They have been associated with tumours in snakes though [143,144]. One of the most commonly reported disease in captive snakes is the inclusion body disease of Boid snakes and although the etiology of the disease is unknown [145], a retroviral infection is often associated [146-149]. The disease is characterised by intracytoplasmic inclusions consisting of a distinctive protein and affected snakes exhibit neurological disorders and regurgitation of food in some species, associated with stomatitis, pneumonia and tumours [150]. The disease may be subclinical, protracted for months or terminal within a few weeks of first clinical signs. Eosinophilic intracytoplasmic inclusion bodies can be present in epithelial cells of all major organs, and meningo-encephalitis is prominent. Supernatant from primary cell cultures of the kidney from an infected boa constrictor (*Boa constrictor*) was inoculated into young Burmese pythons (*Python molurus bivittatus) *with a resultant IBD development [151]. This disease is often found in snakes from collections with severe mite infestations [145].

With the advance of molecular techniques, endogenous retroviruses are identified in many vertebrates, including reptiles [146]. Huder et al. found highly expressed retroviral activity in all individuals of *Python curtus *but not in other boid species, irrespective of them displaying the symptoms of inclusion body disease which further clouds the etiological origins of this disease [146].

### 3.9. Arboviruses

Arboviruses are arthropod borne viruses that multiply in both the arthropod vector and the vertebrate host [152]. Many are pathogenic to humans, but reptiles and amphibians can represent an alternative host in which the virus may overwinter in hibernating reptiles and in some cases produce overt disease [153]. Some flaviviruses, togaviruses, rhabdoviruses and a bunyavirus found in reptiles have been classified as arboviruses.

Chaco, Timbo and Marco rhabdoviruses were isolated from the lizard *Ameiva ameiva *and classified by EM [154]. They grew well in mammalian cell lines with an optimal temperature of 30°C and showed no serological cross-reactivity with other rhabdoviruses [155]. Despite their classification as arboviruses, it still remains unclear if Chaco, Timbo and Marco viruses are able to create sufficient viremia to serve as a source for arthropod infection and the infection was not associated with disease in the reptilian host [156]. Likewise, a virus identified as Bunyamwera was isolated from a turtle (*Trionyx spinifer emoryi*) during a survey of reptiles in Texas in 1970 and 71 [157]. There is no report on the health of the turtle, but the case confirms the role of reptiles as reservoirs for viruses that cause severe disease in humans.

#### 3.9.1. Flaviviridae

Antibodies to St Louis encephalitis virus and Japanese encephalitis virus (JEV) have been reported from turtles and snakes [158-160]. Lee et al. [22] isolated JEV from a Chinese rat snake (*Elaphe rufodorsata*) and in 2001, Drury et al. isolated a flavivirus-like agent from a tortoise [25]. Much of the early evidence for flavivirus infection in reptiles is associated with investigations into their role as reservoirs for this zoonosis, but rarely was the infection reported with disease. When a new strain of West Nile Fever crossed the Atlantic in 1999 and continued across the United States from east to west, much effort went into finding alternative hosts and reservoirs for the virus, focussing the attention back to reptiles among other animals.

Sero-surveys detected antibodies to West Nile Fever virus (WNV) in farmed Nile crocodiles in Israel, farmed crocodiles in Mexico, wild alligators in Florida and free-range American alligators in Louisiana [5,6,153,161]. The virus was found to be associated with disease and mortality in farmed alligators in Georgia, Louisiana and Florida [153,162,163]. Alligators with symptoms in Florida were investigated and a very high load of WNV was detected in the livers with pathological changes in multiple organs [153]. A subsequent transmission study proved the pathogenicity of the isolate to inoculated and cohabiting alligators [7].

#### 3.9.2. Togaviridae

This group of viruses includes members such as Eastern equine encephalitis virus (EEEV), Western equine encephalitis virus (WEEV) and Venezuelan equine encephalitis [152]. Although infection by these viruses appears to be common according to antibody surveys, which detected antibodies against the viruses in several snake, lizard, turtle and one crocodilian species [157,164], there is little evidence of them causing disease in the reptile hosts. Rather, reptiles may function as reservoir hosts due to their low metabolic rate and subsequent reduced immune response in winter [164].

Experimental infection of snakes and tortoises show them to be highly susceptible [1] with viraemia lasting from 3 to 105 days post infection depending on temperature [2]. Hayes et al. found that neutralising antibody persisted for at least 44 days after the viraemia had subsided [1]. Higher ambient temperatures during the experimental trials appeared to raise the titre of antibodies against the virus and reduce the duration of an infection [2].

Isolation of togaviruses from reptiles has been attempted predominantly from blood samples with variable results [152,165]. This may be due to the cyclical nature of viremias [166]. Rosenbusch was able to isolate WEEV from the brain of *Bothrops alternata*, but not from the blood, showing that blood may not be the best organ for viral isolation, and that the virus may replicate in other organs during periods of low or no viremia [21].

One incidence of feeding by an WEEV-infected mosquito (*Culex tarsalis*) was sufficient to transmit the infection to a garter snake [167]. Viremia lasted 70 days post hibernation in snakes that were bitten by infected mosquitoes before hibernation [168]. Conversely, 31% of mosquitoes became infected after feeding on snakes with low level viremia. Vertical transmission between infected mothers and offspring has also been documented for WEEV in garter snakes [165]. A three years survey of blood meals from mosquitoes in an EEEV endemic area in Alabama [169,147] revealed that 75% were from reptiles. This is another example of how a human pathogen can be transmitted to, harboured in, and recovered from reptiles with the aid of an arthropod vector.

### 3.10. Caliciviridae

Sixteen isolates of calicivirus were obtained from four species of poikilothermic animals in a zoological collection [23]. Eight Aruba Island rattlesnakes (*Crotalus unicolor*) were asymptomatic and isolation was obtained by rectal swab. The other eight isolates were obtained at necropsy of animals found dead in their cages. These included four Aruba Island rattlesnakes, two Bell horned frogs (*Ceratophrys orata*), one rock rattlesnake (*C. lepidus*) and one eyelash viper (*Bothrops schlegeli*). Histopathology revealed a variety of inconsistent lesions in the necropsied animals. The isolates grew in Vero cells at 37°C and were identified by physicochemical characteristics as belonging to the Caliciviridae. The 16 isolates were antigenically indistinguishable and the strain was designated as reptilian calicivirus *Crotalus *type 1. These isolates were compared to isolates from feral pinnipeds (San Miguel sea lion calicivirus) and were found to be closely related at the serological and genomic level [170,171], further adding to speculations into how this virus is transmitted between terrestrial reptiles and marine mammals [172].

### 3.11. Picornaviridae

The only record of picornavirus in reptiles is by Helbstad and Bestetti [114]. A boa constrictor with signs of gastrointestinal disease and central nervous system disorder, displayed groups of necrotic cells with intranuclear inclusion bodies throughout the intestinal tract, the liver, pancreas and spleen. Perivascular cuffing was observed in the meninges together with leukoencephalopathy. Adenovirus virions were visualised by EM in the duodenum and spleen, as were picornavirus virions. The latter were small, 22-27 nm diameter, spheroidal and arranged in rows or lattice formation in the cytoplasm of necrotic cells [114]. An Aesculapian snake showed loss of appetite, abnormal faeces and regurgitation. Upon EM examination four different types of viruses were identified in its duodenum, one of which was a picornavirus [114]. In these mixed infections it is difficult to attribute certain pathological changes to a specific virus, and the picornavirus may merely have been an incidental finding of a non-virulent virus. It should however, be noted that other picornaviruses, the human and porcine enteroviruses, manifest first in the alimentary canal, then proceed to the brain, where they can cause encephalitis with subsequent neurological disorders [173].

### 3.12. Paramyxoviridae

Several epizootics in snake collections have been attributed to a paramyxovirus infection. The first record was from Switzerland, with a respiratory disease of farmed Fer-de-lance snakes (*Bothrops atrox*) causing up to 87% mortality in individual rooms [174]. Subsequent outbreaks were reported in rock rattlesnakes (*Crotalus lepidus*) and several viper species [30] as well as non-viper species [175-178]. Paramyxovirus has also been reported from the respiratory tract of lizards with pneumonia [179,180]. Antibody surveys of captive and wild-caught lizards show that they often have elevated antibody titres against paramyxovirus [181-185].

Terminally ill snakes can display neural symptoms [30,186]. However, the target organ seems to be the respiratory tract. Post mortem examination often reveals fluid filled lungs and body cavity [175,187]. Lesions are observed in the lungs and occasionally in the brain. An immuno-histochemical survey of sections from suspected ophidian paramyxovirus infection confirmed that the lungs are the main target organ for the virus and that there is multifocal cytoplasmic staining of infected cells [175].

Virus was isolated from the lungs and brain of infected snakes by propagation in cobra eggs, viper heart, gecko embryo, rattlesnake fibroma and Vero cell lines [29,30,188]. The virus grows better at 28°C than at 37°C, produces syncytia and eventually destroys the cell layer [189].

Electron microscopy of in vitro propagated virus showed the virions to be pleomorphic, spheroidal or filamentous particles budding from plasma membranes or as mature enveloped particles in the cytoplasm [187,188,190]. Paramyxovirus have also been isolated from a Hermann tortoise (*Testudo hermannii*) with pneumonia and identified in faeces of farmed Nile crocodiles [113,191]. However, these virions could potentially have derived from infected chickens fed to the crocodiles, rather than from an active gastrointestinal infection of the crocodiles themselves.

On a genomic level, paramyxoviruses from snakes and lizards are closely related, while the one isolate from the tortoise was in the same cluster but more distant to the other isolates [191]. The same study confirmed that the paramyxoviruses of reptiles are distinct from those isolated from other animals and are genetically sufficiently different from other paramyxoviruses to have their own proposed genus: *Ferlavirus *within the subfamily *Paramyxovirinae *with Fer-de-Lance virus as the type species [16,192].

## 4. Summary

A multitude of viruses exists in reptiles, some of which are described above, and no doubt many more will be described in the future. Some, but not all cause disease and most of them are not very well studied in terms of virulence, pathogenicity, phylogeny and fulfilment of Koch's postulate, which requires cultivation and challenge trials. For viruses like CFPHV that cannot be cultured in vitro, the association with fibropapillomatosis is very strong, but apparently other cofactors are needed before it becomes pathogenic. Transmission may in some cases be via vectors such as arthropods or leeches, whereas others by direct contact presumably, but most transmission routes are unknown. The risk of transfer of viruses between reptiles and humans is negligible due to the thermoregulatory differences between reptilian and mammalian hosts which would limit the suite of pathogens able to grow in both temperature regimes. However, some zoonotic potential exists when certain reptiles act as reservoirs for arboviruses that are pathogenic to humans such as West Nile fever virus.

Compared to fish, poultry and mammalian viruses that are of high importance in our society for either commercial or humanitarian reasons, very little is known about reptilian viruses. Crocodile, turtle and snake farms only exist on a relatively small scale and reptiles still feature as rare and exotic pets although individual specimens may be quite valuable. Specialised veterinary clinics and research scientists take an interest in reptilian viruses though and future directions for research and diagnostic tools are likely to venture further into sero-surveys. Hopefully these will become commercially available, thereby facilitating testing of reptiles prior to transfer between zoological collections to protect valuable specimens from known diseases. As for all other veterinary fields, the future diagnosis of reptilian viruses lies with molecular tools for identification, taxonomy, phylogeny and epidemiology.

## 5. Competing interests

The author declares that they have no competing interests.
